# Fbrsl1 is required for heart development in *Xenopus laevis* and *de novo* variants in *FBRSL1* can cause human heart defects

**DOI:** 10.1242/dmm.050507

**Published:** 2024-05-14

**Authors:** Hanna Berger, Sarah Gerstner, Marc-Frederik Horstmann, Silke Pauli, Annette Borchers

**Affiliations:** ^1^ Department of Biology, Molecular Embryology, Philipps-University Marburg, 35043 Marburg, Germany; ^2^ Institute of Human Genetics, University Medical Center Göttingen, 37073 Göttingen, Germany

**Keywords:** Congenital malformation syndrome, Heart development, Fbrsl1, AUTS2

## Abstract

*De novo* truncating variants in fibrosin-like 1 (*FBRSL1*), a member of the AUTS2 gene family, cause a disability syndrome, including organ malformations such as heart defects. Here, we use *Xenopus laevis* to investigate whether Fbrsl1 plays a role in heart development. *Xenopus laevis fbrsl1* is expressed in tissues relevant for heart development, and morpholino-mediated knockdown of Fbrsl1 results in severely hypoplastic hearts. Our data suggest that Fbrsl1 is required for the development of the first heart field, which contributes to the ventricle and the atria, but not for the second heart field, which gives rise to the outflow tract. The morphant heart phenotype could be rescued using a human N-terminal FBRSL1 isoform that contains an alternative exon, but lacks the AUTS2 domain. N-terminal isoforms carrying patient variants failed to rescue. Interestingly, a long human FBRSL1 isoform, harboring the AUTS2 domain, also did not rescue the morphant heart defects. Thus, our data suggest that different FBRSL1 isoforms may have distinct functions and that only the short N-terminal isoform, appears to be critical for heart development.

## Teaser

**Summary:** Fbrsl1 loss of function causes defects in *Xenopus* heart development. These defects can be rescued with a human FBRSL1 isoform, but not with variants identified in patients with heart defects.

**Research Simplified**

Congenital heart disease (CHD) is a highly prevalent malformation that affects newborns. Patients carrying certain variants of a gene that codes the fibrosin-like 1 (FBRSL1) protein suffer from a rare malformation and intellectual disability syndrome, characterised by a wide spectrum of clinical symptoms including CHD. Understanding whether and how FBRSL1 regulates heart development can help researchers advance therapeutics for FBRSL1-associated CHD.

First, the authors studied the function of FBRSL1 in laboratory frogs as their heart development process is very similar to that in humans. The gene that codes for the FBRSL1 protein localised in tissues important for heart development, indicating the potential role of this gene in frog heart development. Loss of FBRSL1 function showed severe heart defects in some frog embryos, causing incomplete heart development, irregularly shaped heart chambers and disorganised cardiac tissue. These defects could be corrected by introducing a short isoform of human FBRSL1 that comprises only the first part of the protein into the frog embryos lacking functional FBRSL1.

This study uncovered that FBRSL1 plays a critical role during heart development in frogs – specifically in the formation of proper heart chambers. Further investigation into how distinct FBRSL1 variants cause CHDs in humans can potentially accelerate the discovery of novel therapeutics for rare heart defects.

## INTRODUCTION

We recently identified truncating variants in the *FBRSL1* gene in three unrelated children who showed strikingly similar malformations (Pauli et al., 2021; Ufartes et al., 2020). The patients presented with facial dysmorphism, cleft palate, microcephaly, skeletal anomalies and contractures, skin creases, developmental delay, and growth retardation. Respiratory problems, hearing impairment and heart defects were also observed. All variants cluster in the N-terminal region of the FBRSL1 protein encoded by exons 2 and 3, and lead to premature stop codons, whereby the severity of the malformations increases with the distance of the variant from the ATG region. Two severely affected children, carrying either the *FBRSL1* variant c.487C>T (p.Q163*) or the deletion c.581_603del, were diagnosed at birth, when they showed respiratory and feeding problems in combination with craniofacial anomalies and excessive skin folds at the arms, legs and back (Ufartes et al., 2020). Furthermore, these children also had heart defects: one was an atrial septal defect (ASD) in combination with a persistent ductus arteriosus, the other was an atrial and ventricular septal defect (VSD) (Ufartes et al., 2020). Thus, FBRSL1 likely plays distinct roles during embryonic development.

The function of FBRSL1 in embryonic development has so far not been well characterized. In zebrafish, *fbrsl1* is expressed in the developing brain, the spinal cord, the cranial ganglia and the somites (Kondrychyn et al., 2017). In *Xenopus laevis*, we have shown that *fbrsl1* is expressed throughout early development (Ufartes et al., 2020). At tailbud stages, *fbrsl1* transcripts are detected in the branchial arches, the cranial nerves and the brain. Furthermore, morpholino-mediated knockdown of Fbrsl1 resulted in craniofacial defects and a reduction in brain size on the injected side. In addition, the migration of cranial and motor neurons was impaired (Ufartes et al., 2020). As patients carrying *FBRSL1* variants also have heart defects, it is likely that Fbrsl1 plays a role in heart development.

Vertebrate heart development is an evolutionarily conserved process that can be well studied using the *Xenopus* system (Duncan and Khokha, 2016; Hempel and Kühl, 2016; Hoppler and Conlon, 2020; Kaltenbrun et al., 2011; Kostiuk and Khokha, 2021; Warkman and Krieg, 2007). Advantages include the rapid external development of embryos, established techniques for tissue-specific micromanipulation and the fact that embryos can survive to early tadpole stages without a functional circulatory system (Hempel and Kühl, 2016; Hoppler and Conlon, 2020). The *Xenopus* three-chambered heart consists of two atria and a single ventricle and represents an intermediate between the two-chambered heart of the fish and the four-chambered heart of birds and mammals (Gessert and Kühl, 2009). *Xenopus* cardiogenesis begins at gastrulation, when the precardiac mesoderm forms on the dorsal side, adjacent to both sides of the Spemann's organizer (Nascone and Mercola, 1995; Sater and Jacobson, 1989, 1990). During gastrulation, these cells move anterior to the ventral midline, where they fuse to form a crescent-shaped structure that will divide into the first heart field (FHF) and the second heart field (SHF) (Gessert and Kühl, 2009). The *Xenopus* FHF will give rise to the ventricle and the two atria, while the SHF will form the outflow tract. In contrast to amniotes, outflow tract septation in *Xenopus* relies solely on the second heart field and not on the cardiac neural crest cells (Lee and Saint-Jeannet, 2011). Key stages of heart development have been defined by comparative analysis of the spatio-temporal expression pattern of cardiac marker genes and morphological features (Gessert and Kühl, 2009; Kolker et al., 2000; Mohun et al., 2000). These can now serve as reference points for functional analysis. Here, we use the *Xenopus* system to analyze the function of Fbrsl1 in heart development.

## RESULTS

### *Fbrsl1* is expressed in the developing *Xenopus* heart

**Fig. 1.**
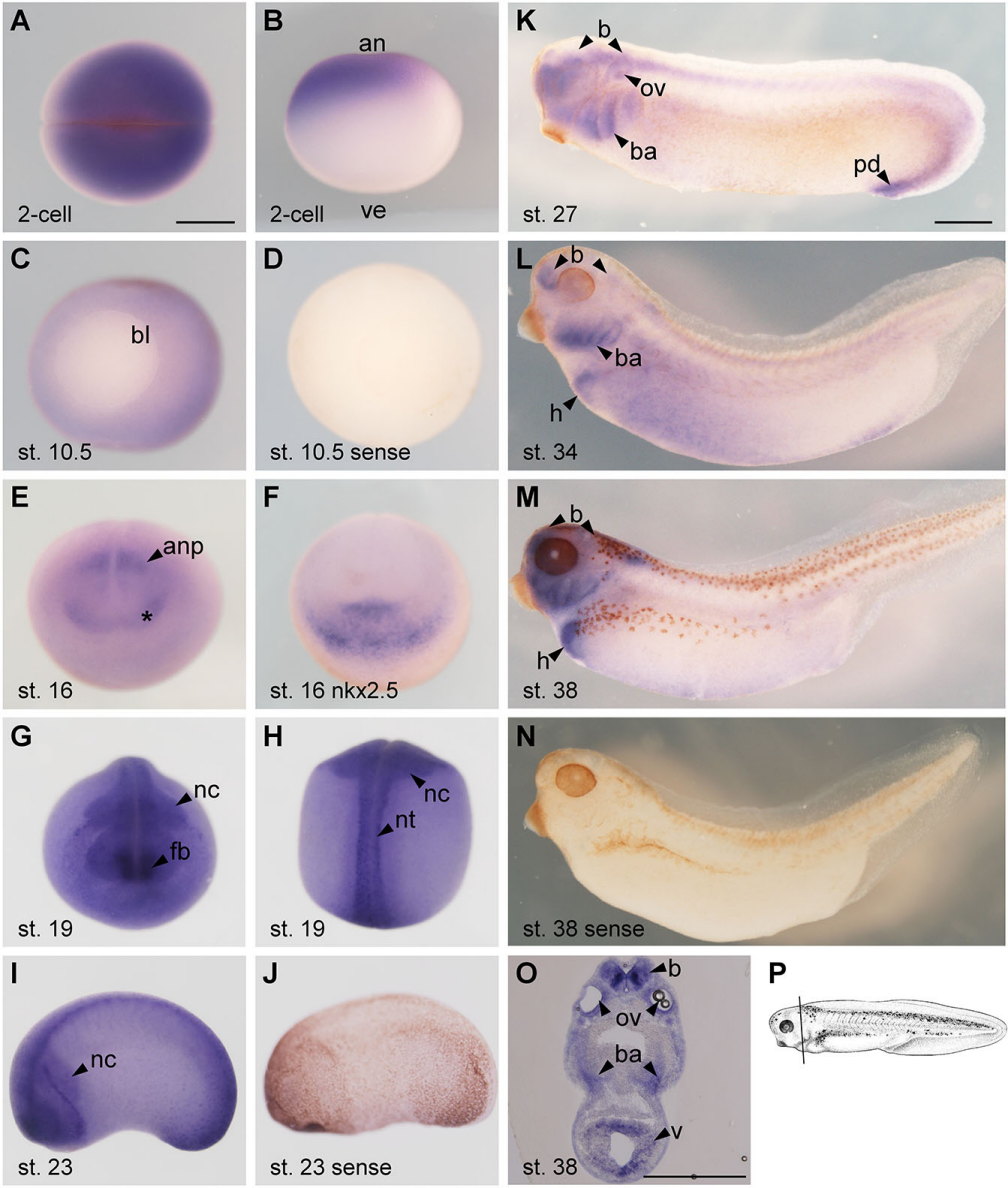
***Xenopus fbrsl1* spatial expression pattern analyzed by whole-mount *in situ* hybridization.** (A,B) An embryo at the two-cell stage: animal view (A); lateral view (B). (C) Embryo at the blastula stage. (D) Embryo at blastula stage, hybridized with the *fbrsl1* sense probe. (E) Embryo at the neurula stage, anterior view. Asterisk indicates the crescent-shaped expression domain. (F) Embryo at the neurula stage stained using a *nkx2.5* antisense probe, anterior view. (G,H) An embryo at stage 19: anterior view (G); dorsal view (H). (I) Embryo at stage 23, lateral view. (J) Embryo at stage 23 hybridized using the *fbrsl1* sense probe. (K) Embryo at stage 27, lateral view. (L) Embryo at stage 34, lateral view. (M) Embryo at stage 38, lateral view. (N) Embryo at stage 38, lateral view, hybridized with the *fbrsl1* sense probe. (O) Transverse section of an embryo at stage 38. (P) Schematic indicating the section plane of the stage 38 embryo (Zahn et al., 2022) in O. Scale bars: 500 µm. an, animal; anp, anterior neural plate; b, brain; ba, branchial arches; bl, blastoporus; fb, forebrain; h, heart; nc, neural crest; nt, neural tube; ov, otic vesicle; pd, proctodeum; v, ventricle; ve, vegetal.

In order to study the function of Fbrsl1 in *Xenopus* heart development, we first analyzed the *fbrsl1* expression pattern using an antisense probe detecting *Xenopus laevis* full-length *fbrsl1*. Consistent with our previously published temporal *fbrsl1* expression pattern analyzed by RT-PCR (Ufartes et al., 2020), whole-mount *in situ* hybridization demonstrated *fbrsl1* expression at all stages analyzed, starting from fertilized oocytes to tailbud stages (Fig. 1). Maternal *fbrsl1* expression was detected in the animal pole at stage 2 (Fig. 1A,B), followed by a broad *fbrsl1* expression during gastrulation, with the exception of the blastoporus (Fig. 1C). No staining was observed using the full-length *fbrsl1* sense probe as a control (Fig. 1D). At early neurula stages, *fbrsl1* expression was detected in the anterior neural plate, whereas this area was not stained using the *fbrsl1* sense control (Fig. 1E; Fig. S1). At these stages, the cardiac progenitor cells localize in an area that encompasses the crescent-shaped *fbrsl1*-expressing cells at the anterior end. For example, *nkx2.5* (Fig. 1F) is expressed in an anterior domain (Gessert and Kühl, 2009) covering the area of the *fbrsl1*-expressing cells. However, at neurula stages, where cardiac markers show a distinct expression domain ventral to the cement gland (Gessert and Kühl, 2009), *fbrsl1* expression is not detected in this domain*.* Nevertheless, there is a general enrichment of *fbrsl1* expression in the anterior region of the embryo, which could suggest it has a function in early heart development. *fbrsl1* also continues to be expressed in the closed neural tube, with enrichment in the future brain area. Furthermore, *fbrsl1* expression is detected in migratory cranial neural crest cells (Fig. 1G-I; Fig. 1J, sense control). At tailbud stages, *fbrsl1* is expressed in the neural tube, the branchial arches, the brain, the otic vesicle and the proctodeum (Fig. 1K,L). Consistent with a potential role in heart development, *fbrsl1* expression is detected in the heart at late tailbud stages (Fig. 1L). Heart expression is also seen at free-swimming tadpole stages, but is not detected in the *fbrsl1* sense controls (Fig. 1M,N). Transverse sections confirm that *fbrsl1* is expressed in the ventricle of free-swimming tadpoles (Fig. 1O,P). In addition, free-swimming tadpoles show *fbrsl1* expression in the branchial arches and the brain (Fig. 1M). Taken together, the *fbrsl1* spatial expression pattern is consistent with a potential function in *Xenopus* heart development.

### Fbrsl1 loss of function causes defects in heart development

**Fig. 2.**
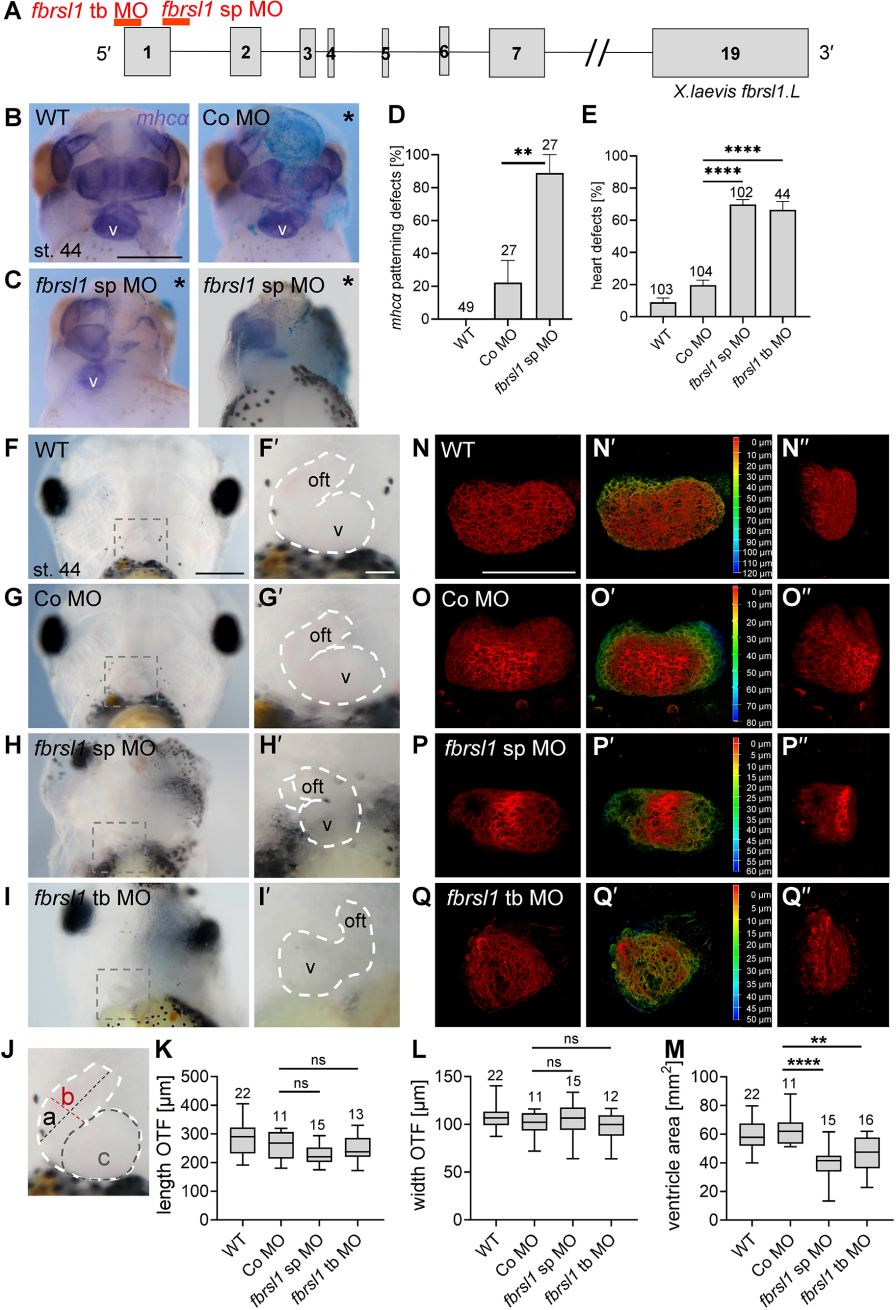
**Fbrsl1 loss of function leads to cardiac defects in *Xenopus* embryos.** (A) Schematic showing morpholino-binding sites at the 5′ region of *Xenopus laevis fbrsl1* (full-length *X. laevis fbrsl1* consists of 19 exons). The *fbrsl1* translation blocking (tb) MO targets the translational start site, whereas the *fbrsl1* splice blocking (sp) MO targets the exon 1/intron 1 boundary (Ufartes et al., 2020). (B,C) Embryos were injected with either 10 ng control (Co) MO or 7.5 ng *fbrsl1* sp MO together with 100 pg *lacZ* RNA as lineage tracer in one dorsal blastomere at the four-cell stage. *mhcα* (*myh6*) *in situ* hybridization marks the heart and facial muscles in wild-type and morpholino-injected embryos. (B) Wild-type and Co MO-injected embryos. (C) Embryos injected with *fbrsl1* sp MO. Scale bar: 500 µm. (D) Graph summarizing *mhcα* heart patterning defects of three independent experiments. Data are mean±s.e.m.; the numbers of embryos are indicated, **P=0.0041 (one-way ANOVA with Tukey's post hoc test). (E) Embryos were injected with 7.5 ng morpholino oligonucleotides in one dorsal blastomere at the four-cell stage, combined with 50 pg *mGFP* RNA (CT3) or 100 pg *lacZ* RNA (MF20) as lineage tracer. Graph summarizing heart defects of CT3- or MF20-immunostained embryos from at least three independent experiments. Data are mean±s.e.m.; the numbers of embryos analyzed (*n*) are indicated, ****P<0.0001 (one-way ANOVA with Tukey's post hoc test). (F-I) Four-cell stage embryos were injected in one dorsal blastomere with 7.5 ng of the respective morpholino oligonucleotides in combination with 50 pg *mGFP* RNA as lineage tracers. Heart morphology was analyzed at stage 44; embryos are shown from the ventral side. (F′-I′) Higher magnification of the areas outlined in F-I. (F-G′) Wild-type (F,F′) and Co MO-injected (G,G′) embryos show normal heart morphology. Ventricle (v) and outflow tract (oft) are marked by dashed white lines. (H-I′) Injection of either the *fbrsl1* sp MO (H,H′) or the *fbrsl1* tb MO (I,I′) leads to defects in heart morphology. (N-Q) *Z*-stack images of *Xenopus* hearts immunostained for cardiac muscle troponin (CT3). (N,O) The heart ventricle is oval in wild-type (N) or Co MO-injected (O) embryos. (P,Q) Heart shape and morphology are disturbed after injection of *fbrsl1* sp MO (P) or the *fbrsl1* tb MO (Q). (N′-Q′) Depth color-coding profile indicates the extension of the heart ventricles from 0 µm (red) to 50-120 µm (blue). The endpoints are different in N′-Q′. (N″-Q″) *xz* views of heart ventricles. (J-M) Embryos from the experiments shown in F-I were used to measure the length of the OTF (a), the width of the OFT (b) and the ventricular area (c), as indicated in J. (K-M) Box and whiskers plots summarize the length (K) and the width (L) of the OFT as well as the ventricular area (M). The number of hearts measured and the median are indicated. The boxes extend from the 25th to the 75th percentiles with whiskers ranging from minimum to maximum values. ns, not significant. **P<0.01, ****P<0.0001 (one-way ANOVA with Tukey's post hoc test). Scale bars: 500 µm in F-I; 100 µm in F′-I′; 200 µm in N-Q″.

To analyze whether Fbrsl1 loss of function affects heart morphology, we used loss-of-function studies. To this end, two distinct morpholino oligonucleotides were used to knockdown Fbrsl1 in *Xenopus laevis* embryos: first, we used the previously published *fbrsl1* splice-blocking morpholino (Ufartes et al., 2020), hereafter referred to as *fbrsl1* sp MO; and second, we used a *fbrsl1* translation blocking morpholino, hereafter referred to as *fbrsl1* tb MO (Fig. 2A). The splice-blocking morpholino (*fbrsl1* sp MO) blocks splicing at the exon 1/intron 1 boundary, leading to the inclusion of intron 1 and the generation of novel in-frame stop codons, resulting in a severe truncation of the protein (Ufartes et al., 2020). The effectiveness of the *fbrsl1* tb MO was verified by western blot analysis (Fig. S2) showing that it inhibits the translation of a GFP-labeled *Xenopus laevis* fbrsl1-transcript containing the *fbrsl1* tb MO-binding site. The different morpholino oligonucleotides were unilaterally targeted to the cardiac mesoderm and heart morphology was analyzed at tadpole stages. This strategy allows the direct comparison of the injected and uninjected (control) side in a single embryo, which is advantageous for analyzing early stages of heart development, where markers are bilateral symmetrically expressed. The injected embryos were analyzed by *in situ* hybridization for *mhcα* (α-myosin heavy chain, *myh6*), which is expressed in the myocardium, including the ventricle, atria and outflow tract, as well as the jaw muscles (Garriock et al., 2005). Wild-type embryos had no defects (Fig. 2B, left panel; Fig. 2D) and distinct structures of the heart could be distinguished. Similar to wild-type embryos, control MO-injected embryos showed only a few minor defects (Fig. 2B, right panel; Fig. 2D). In contrast, *fbrsl1* morphant embryos showed severe defects in heart formation, which ranged from hypoplastic hearts to the complete absence of the heart (Fig. 2C,D). In addition, heart defects were quantified at tadpole stages by fluorescent imaging of embryos immunostained for cardiac muscle troponin T (CT3) or myosin heavy chain (MF20) (Fig. S3), confirming the data obtained by *in situ* hybridization for *mhcα* (Fig. 2E).

Next, live imaging was used to further analyze beating heart morphology in free-swimming tadpole embryos. Whereas wild-type or control morpholino (Co MO)-injected embryos showed a normal heart morphology, (Fig. 2F,G, Movies 1 and 2), depletion of Fbrsl1 by either the *fbrsl1* sp MO or the *fbrsl1* tb MO resulted in severe hypoplasia of the heart (Fig. 2H,I, Movies 3 and 4). Interestingly, we observed that the outflow tracts were the least affected structure in the morphant hearts, whereas the ventricular defects appeared more severe. To test whether this observation is correct, we used live-imaging of injected and wild-type tadpole embryos, and measured the length and the width of the outflow tract as well as the area of the ventricle at the time point of contraction (Fig. 2J). Indeed, the length and width of the outflow tract was not significantly affected (Fig. 2K,L), whereas the ventricle area decreased in *fbrsl1* morphant embryos (Fig. 2M).

To further asses ventricle morphology in detail, high-resolution fluorescence microscopy of CT3 stained tadpole embryos was used (Fig. 2N-Q). Z-stacks were recorded and the depth of the z-stacks was visualized using a color-code. Whereas wild-type and control MO-injected embryos (Fig. 2N,O) showed a regular oval-shaped ventricle, Fbrsl1 loss of function caused severe defects in ventricle formation (Fig. 2P,Q). The ventricles of *fbrsl1* morphant embryos were smaller and irregularly shaped, and the cardiac tissue appeared severely disorganized (Fig. 2P,Q). Taken together, and in light of the findings that variants in *FBRSL1* in humans cause heart defects (Ufartes et al., 2020), these data support a function for Fbrsl1 in heart development.

### Fbrsl1 is not required for cardiac mesoderm induction, but for the formation of the first heart field

**Fig. 3.**
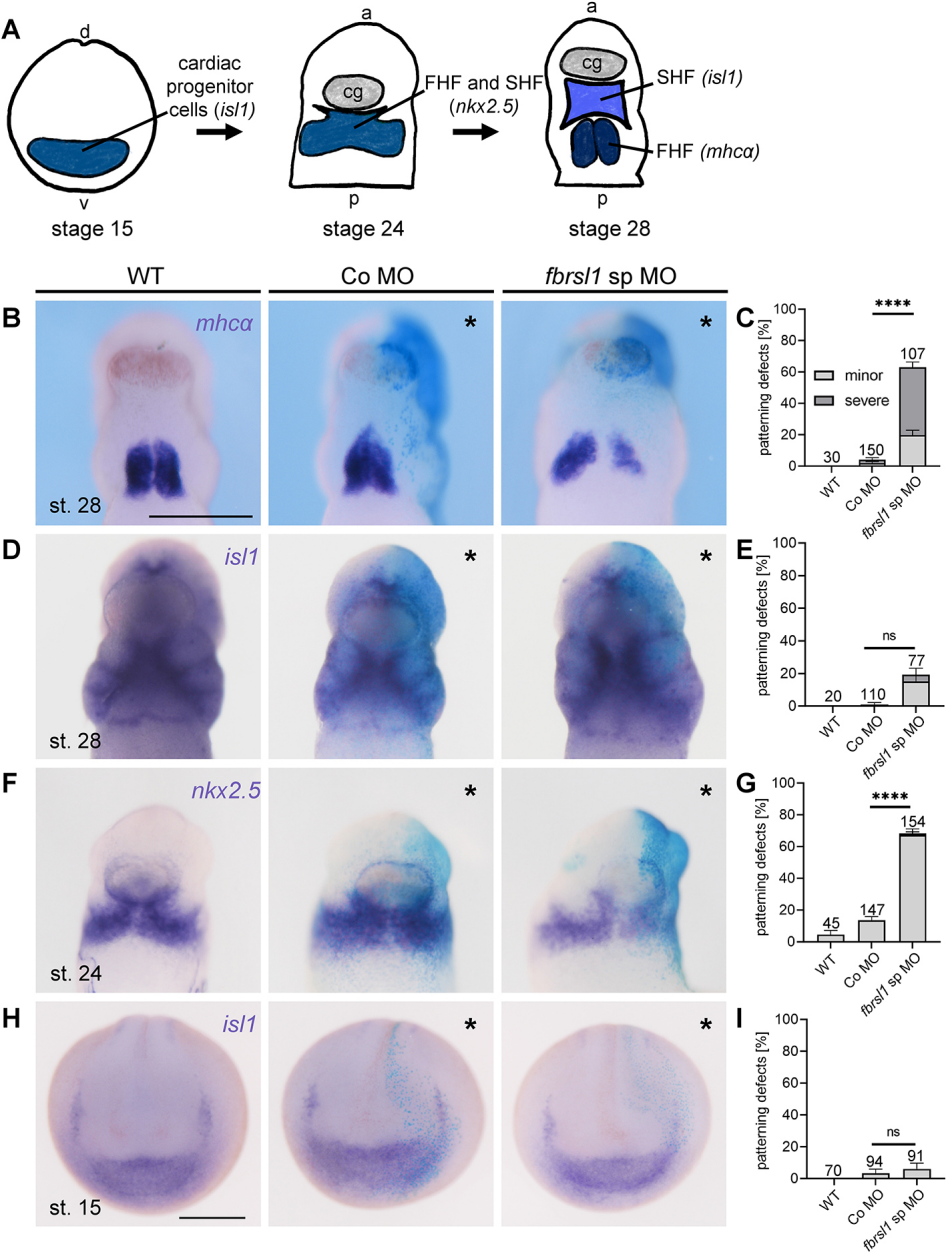
**Fbrsl1 loss of function disrupts the development of the first heart field, but not the cardiac progenitor cells.** (A) Schematic illustration of the key steps in *Xenopus* heart development. At stage 15, cardiac progenitor cells are located anterior in a crescent-like structure (marked by *isl1* expression), which gives rise to two separate populations: the first heart field (FHF) and the second heart field (SHF). At stage 24, *nkx2.5* is expressed in both heart fields, whereas at stage 28 the two cell populations can be distinguished using the markers *mhcα* (FHF) and *isl1* (SHF). (B-I) Embryos were injected at the four-cell stage into one dorsal blastomere with 5 ng-7.5 ng (B-G) or 7.5 ng-10 ng (H,I) control (Co) MO or *fbrsl1* splice blocking (sp) MO and 80 pg *lacZ* RNA for lineage tracing, cultured to the respective stages and analyzed by *in situ* hybridization. Asterisks indicate the injected side. Scale bar: 500 µm. Embryos are shown from the ventral (B-F) or the anterior (H) side. (B) Stage 28 embryos analyzed by *mhcα* whole-mount *in situ* hybridization. *fbrsl1* sp MO-injected embryos show a reduction of the first heart field on the injected side (minor defect is shown). (C) Graph summarizing the defects in first heart field formation of three independent experiments. (D) Stage 28 embryos analyzed by *isl1 in situ* hybridization. Expression is visible in the second heart field. The *fbrsl1* sp MO-injected side shows normal expression. (E) Graph summarizing the defects in second heart field formation of two (wild type) or three independent experiments. (F) Embryos analyzed by *nkx2.5* whole-mount *in situ* hybridization at stage 24. Expression can be observed in the first and second heart field. The *fbrsl1* sp MO-injected side shows reduced expression (minor defect is shown). (G) Graph summarizing the results of three independent experiments shown in F. (H) Whole-mount *in situ* hybridization showing *isl1* expression in the cardiac progenitor cells. The *fbrsl1* sp MO-injected side shows normal expression. (I) Graph summarizing the defects of cardiac progenitor cells of three independent experiments. Data are mean±s.e.m. The numbers of evaluated embryos are indicated in all graphs. ns, not significant. ****P<0.0001 (one-way ANOVA with Tukey's post hoc test). a, anterior; d, dorsal; p, posterior; v, ventral.

To determine at which stages of heart development Fbrsl1 is required, we analyzed cardiac marker expression at different stages of heart development (Fig. 3A). We started our analysis at the tailbud stage to determine whether *fbrsl1* loss of function affects cardiac differentiation, which was again assessed by *mhcα* expression, as a marker for the earliest stages of cardiac differentiation. Consistent with the severe defects seen at tadpole stages, the morphant embryos showed a strong reduction in *mhcα* expression on the injected side compared with the control side, whereas control embryos looked normal (Fig. 3B,C; Fig. S4). Thus, cardiac differentiation appears to be impaired by Fbrsl1 loss of function. Furthermore, these data indicate that the development of the first heart field is inhibited, as *mhcα* is expressed at these stages in the first heart field (Gessert and Kühl, 2009). Next, we analyzed the development of the second heart field by performing *islet* 1 (*isl1*) *in situ* hybridization, which marks the second heart field (Brade et al., 2007). Here, we did not observe any significant defects (Fig. 3D,E; Fig. S4), suggesting that the development of the second heart field is not affected. To determine whether Fbrsl1 is also involved in earlier stages of heart development, we analyzed the expression of *nkx2.5*, a transcription factor that marks the development of the first and second heart field (Brade et al., 2007; Newman and Krieg, 1998). We observed a significant reduction in *nkx2.5*. expression on the injected side (Fig. 3F,G), indicating a role in lineage specification of the first and second heart fields. Finally, we used *isl1 in situ* hybridization to assess the development of the precardiac precursors. Interestingly, we did not observe any defects, suggesting that this early step in heart development is not affected by loss of Fbrsl1 function (Fig. 3H,I). These data suggest that Fbrsl1 is required for the development of the first heart field, which is relevant for the development of the ventricle and the two atria.

### The short N-terminal isoform of FBRSL1 is relevant for heart development

**Fig. 4.**
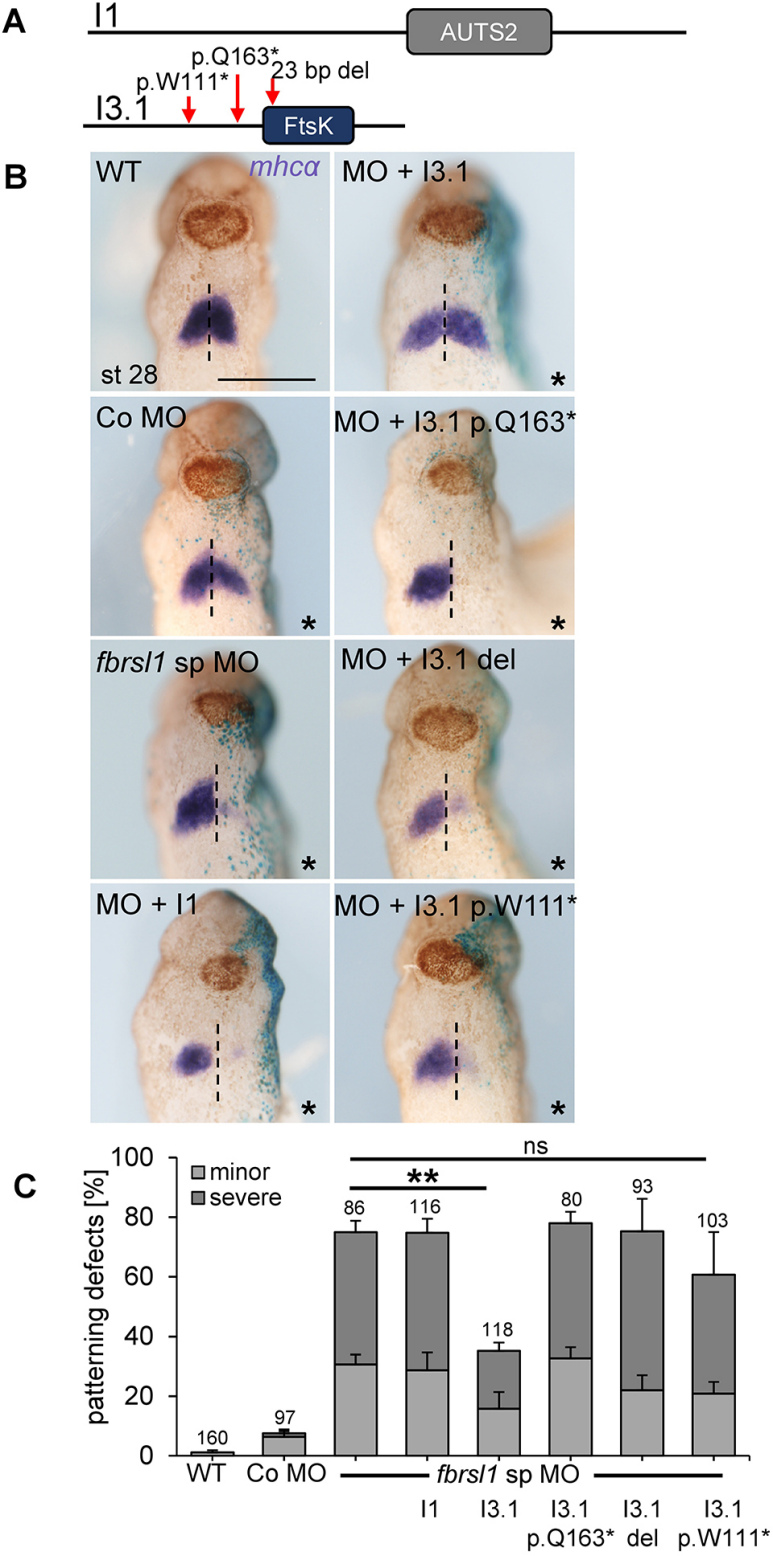
**Defects in *mhcα* patterning are rescued by the human short N-terminal FBRSL1 isoform 3.1.** (A) Schematic of the FBRSL1 isoform 1 and the short N-terminal isoform 3.1. Red arrows indicate the position of the three patient variants. (B) 7.5 ng of the respective morpholino oligonucleotides and 150 pg of the plasmids were injected in one dorsal blastomere at the four-cell stage. 100 pg *lacZ* RNA was co-injected as lineage tracer. Representative embryos at stage 28 are shown from the ventral side; injected constructs are indicated. Asterisks indicate the injected side; blue β-galactosidase staining is visible. Scale bar: 500 µm. (C) The graph summarizes the percentage of *mhcα* patterning defects of three independent experiments. Data are mean±s.e.m. The numbers of embryos analyzed are indicated. **P<0.01; ns, not significant (*P*=0.06) (one-way ANOVA with Tukey's post hoc test).

To determine whether the observed defects of *fbrsl1* morphants were specific to loss of Fbrsl1 function, we performed rescue experiments. We recently showed that our patients carried variants in a short N-terminal isoform of *FBRSL1*, which lacks the AUTS2 family domain, but includes a predicted DNA translocase domain (Ftsk) (NCBI conserved database, CDD) (Ufartes et al., 2020). The position of the identified variants (p.W111, p.Q163) as well as a 23 bp deletion (c.581_603del) are indicated in Fig. 4A. For rescue experiments, morpholino oligonucleotides alone or in combination with the respective FBRSL1 plasmids, were injected into one blastomere at the four-cell stage and heart development was analyzed at tailbud stages by *mhcα* whole-mount *in situ* hybridization. As expected, injection of the *fbrsl1* sp MO significantly reduced *mhcα* expression on the injected side of the embryo*,* whereas wild-type or Co MO-injected embryos showed the typical *mhcα* expression in the first heart field (Fig. 4B,C). Co-injection of the long human FBRSL1 isoform I1, which contains the AUTS2 domain, but lacks the alternative exon 3 encoded by the short N-terminal isoform I3.1 (Fig. 4A), was not able to rescue the *fbrsl1* morphant phenotype. In contrast, co-injection of the short N-terminal isoform 3.1 (I3.1) significantly rescued the *mhcα* patterning defects caused by Fbrsl1 depletion. However, co-injection of the human FBRSL1 isoform 3.1 (3.I1) carrying the respective patient variants I3.1-p.Q163*, I3.1-p.W111* and I3.1-del (c.581_603del) were not able to rescue the *mhcα* patterning defects (Fig. 4B,C). Thus, these data indicate that the N-terminal isoform, which carries the alternative exon 3, is relevant for heart development and that the patient variants indeed compromise this function.

## DISCUSSION

Congenital heart disease (CHD) is the most common congenital malformation, affecting ∼1% of newborns each year. More than 400 genes have been implicated in the development of CHD, including transcription factors, chromatin remodelers/modifiers, ciliary genes, myofilament and extracellular matrix genes, and genes involved in various signaling cascades (e.g. RAS, Notch and Wnt signaling) (Williams et al., 2019). We have recently described a congenital malformation syndrome, caused by heterozygous truncating variants in *FBRSL1*. Two of the three patients presented with, in addition to other malformations, a congenital heart defect. One patient has a hemodynamically relevant ASD, whereas the other was born with ASD and VSD. A highly variable cardiac phenotype, ranging from mild to severe disease, has been described for various malformation syndromes (e.g. CHARGE syndrome) (Meisner and Martin, 2020). However, owing to the small number of patients with FBRSL1-associated syndrome, it is not yet possible to statistically evaluate the range of possible associated heart defects. As heart defects have a significant impact on the clinical outcome of these patients, we have focused our scientific studies on this clinical aspect.

We used the *Xenopus* system to shed light into the function of FBRSL1 in heart development and to test whether the patient variants are critically affecting function. We find evidence that Fbrsl1 is required for the development of the first heart field that will later give rise to the ventricle and the atria, which is consistent with the ASD and VSD observed in the patients carrying *FBRSL1* variants. Consistently, *fbrsl1* morphants showed malformations as well as a reduction in the size of the ventricle at tadpole stages. Interestingly, we did not observe any defects in the formation of the *Xenopus* second heart field, nor did we see significant defects in the formation of the outflow tract. In contrast to amniotes, *Xenopus* cardiac neural crest cells do not appear to migrate into the cardiac cushion or to contribute to the septum of the outflow tract (Lee and Saint-Jeannet, 2011). Therefore, we cannot rule out the possibility that defects in neural crest migration may also contribute to the clinical phenotype. A role for Fbrsl1 in neural crest development seems likely, because *fbrsl1* morphant embryos show severe craniofacial defects (Ufartes et al., 2020). However, future research is needed to elucidate this role and its implications for heart development.

To date, it is unclear whether the *FBRSL1* variants in patients exert their effect through a loss-of-function or a dominant-negative effect. For our *Xenopus* studies, we used morpholino oligonucleotide injections to knock down Fbrsl1 protein expression. As we used different embryonic batches, which are also genetically variable, the effectiveness of this strategy will vary from embryo to embryo. This may explain why some of the embryos showed a complete loss of heart structures, whereas the defects were less severe in others. This is also supported by the finding that embryos lacking heart structures also showed severe defects in craniofacial development. In addition, the severity of the defects also seemed to increase over time. Although the induction of the cardiogenic mesoderm is not affected by Fbrsl1 knockdown, the severity of defects increased from early to later tailbud stages, suggesting that Fbrsl1 plays a role during subsequent stages of heart development.

To test which isoforms of FBRSL1 are required and whether the patient variants are critically affecting function, we performed rescue experiments. In contrast to AUTS2 syndrome, where the C terminus containing the AUTS2 domain is mostly relevant (Beunders et al., 2013), the situation is different for the FBRSL1-associated syndrome. Here, only the short human N-terminal FBRSL1 isoform containing an alternative exon 3, which includes a stop codon and thus results in a short protein consisting of only three exons, was able to rescue the developmental heart defects of *fbrsl1* morphant *Xenopus* embryos. This was not possible using the long human FBRSL1 isoform harboring the AUTS2 domain, but lacking exon 3. These findings are also consistent with our previous data showing that the human N-terminal isoform of FBRSL1 – but not the long isoform – rescued *Xenopus* morphant craniofacial defects, indicating that this isoform is relevant for embryonic development. Currently, we do not know whether only the N-terminal isoform of FBRSL1 is relevant for embryonic development or whether this only holds true for heart and craniofacial development. In particular, considering the importance of AUTS2 for neural development and autism related syndromes (Biel et al., 2022; Pang et al., 2021), the FBRSL1 long isoform, which contains an AUTS2 domain, potentially also plays a role in neural development, which we know is also affected in *fbrsl1* morphants (Ufartes et al., 2020).

The cellular function of the *FBRSL1* gene and its role in pathogenesis are largely unknown. However, as FBRSL1 and AUTS2 are paralogs, they likely share common conserved functions, which may contribute to the overlapping phenotypes observed in the respective syndromes. FBRSL1 and AUTS2 form – together with fibrosin (FBRS) – the AUTS2 tripartite gene family (Singh et al., 2015). The members of this protein family share conserved domains, but also have unique regions that likely contribute to their distinct functions (Sellers et al., 2020). In neurons, a dual function of AUTS2 has been described (Hori and Hoshino, 2017). In the nucleus, it regulates gene transcription as a component of the Polycomb repressive complex (PRC). In addition, it also affects cytoskeletal dynamics by regulating small GTPases of the Rho family (Gao et al., 2014; Hori et al., 2014). For example, AUTS2 activates Rac1 to induce lamellipodia, while it suppresses filopodia formation by downregulating Cdc42 (Hori et al., 2014). Nuclear and cytoplasmic AUTS2 functions are controlled by different domains of the AUTS2 protein: the cytoplasmic function resides in the N-terminal region of the AUTS2 protein, whereas the interaction with the PRC complex is mediated by the C-terminal region (Bedogni et al., 2010; Hori et al., 2014; Oksenberg and Ahituv, 2013; Sellers et al., 2020). Both AUTS2 and FBRSL1 are components of the PRC1.3 and PRC1.5 complexes (Chittock et al., 2017; Gao et al., 2014). The multiprotein PRC complexes act as epigenetic regulators during embryonic development and function as transcriptional repressors. PRC complexes have also been described as transcriptional regulators in heart development (Akerberg and Pu, 2020). Using the *Xenopus* model system, we observed a significant reduction in *nkx2.5* expression in *fbrsl1* morphants. *NKX2.5* is a cardiac transcription factor and heterozygous pathogenic variants lead to congenital heart defects in humans, such as ASD and VSD (Schott et al., 1998). Further studies are required to analyze whether the observed reduction in *nkx2.5* expression is due to a transcriptional misregulation involving the PRC complex or other pathways such as Rac1 or Cdc42 signaling. Interestingly in humans, pathogenic variants in both *RAC1* and *CDC42* have been associated with malformation syndromes, including congenital heart defects. Pathogenic variants in *RAC1* cause the autosomal dominant inherited intellectual developmental disorder type 48 (OMIM # 617751), while heterozygous pathogenic variants in *CDC42* can cause Takenouchi-Kosaki syndrome (OMIM # 616737). Patients with atrial and/or ventricular septal defects have been described in both syndromes (Martinelli et al., 2018; Reijnders et al., 2017; Szczawińska-Popłonyk et al., 2023). Furthermore, Rac1 deficiency in murine neonatal cardiomyocytes leads to defects in lamellipodia formation, cell elongation and polarity, as well as in increased apoptosis and reduced expression of the cardiac transcription factors Gata4, Tbx5, Nkx2.5 and Hand2 (Leung et al., 2014). Thus, although our data show that Fbrsl1 is required for heart development and *FBRSL1* variants negatively affect this process, future research is required to address the genetics and cellular mechanism by which the variants exert their pathogenic role.

## MATERIALS AND METHODS

### *Xenopus* microinjection

All procedures involving *Xenopus* embryos were performed according to the German animal use and care law (Tierschutzgesetz) and approved by the German state administration Hesse (Regierungspräsidium Giessen, V 7/2022). *Xenopus* laevis embryos were obtained and cultured following standard protocols, and staged according to the normal table of Nieuwkoop and Faber (Nieuwkoop and Faber, 1956). For microinjection, capped sense RNA was synthetized using the mMessage mMachine SP6 Transcription Kit (Invitrogen). The following plasmids were used for *in vitro* transcription: lacZ (Smith and Harland, 1991), mGFP (Moriyoshi et al., 1996) and *X. laevis* GFP-tagged Fbrsl1 isoform A (Fbrsl1_A-eGFP). The *Xenopus laevis fbrsl1* isoform A (2028 bp) is identical to full-length Fbrsl1, but lacks exon 2 and 3, and most of exon 19. For cloning of Fbrsl1_A-eGFP, *fbrsl1_A* was amplified by PCR from *Xenopus laevis* cDNA using the primers 5′-ATGGATATTAAAACCAAACAACCAAGCAGG-3′ and 5′-TATCGTGCCTCCACTTCCTTAGGG-3′. The PCR product was cloned into the pCR-Blunt II-TOPO vector (Zero Blunt TOPO™ PCR cloning kit, ThermoFisher Scientific) and subsequently subcloned into the pCS2+-eGFP vector using the EcoRI and XhoI restriction sites. The following morpholino oligonucleotides (MO) (Gene Tools) were used for microinjections: Standard control morpholino (Co MO: 5′-CCTCTTACCTCAGTTACAATTTATA-3′), *fbrsl1* sp MO (Ufartes et al., 2020) and *fbrsl1* tb MO (*fbrsl1* tb: 5′-GGTTGTTTGGTTTTAATATCCATCT-3′). Both *fbrsl1* sp MO and *fbrsl1* tb MO, target *fbrsl1.L* and show one mismatch (*fbrsl1* sp MO) or four mismatches (*fbrsl1* tb MO) to *fbrsl1.S* (Fig. S5). For rescue experiments, plasmids encoding isoform 1, isoform 3.1 and the variant isoform 3.1-p.Q163* (Ufartes et al., 2020) were co-injected with the *fbrsl1* sp MO. In addition, the non-sense variant, c.332 G>A (p.W111*) was introduced by site-directed mutagenesis of variant 3.1 using the QuikChange II XL Site-Directed Mutagenesis Kit (Agilent) according to the manufacturer's protocol and verified by Sanger sequencing. The construct containing the deletion c.581_603del was generated using patient cDNA. The resulting plasmids were also used for rescue experiments.

### Whole-mount immunofluorescence staining of *Xenopus* embryos

*Xenopus laevis* embryos were injected with Co MO, *fbrsl1* sp MO or *fbrsl1* tb MO in one dorsal blastomere at the four-cell stage, combined with *mGFP* RNA (CT3) or *lacZ* RNA (MF20) as a lineage tracer. The embryos were incubated until stage 44, then they were fixed with Dent's fixative (20% DMSO and 80% methanol, for CT3) or MEMFA (3.7% formaldehyde, 0.1 M MOPS, 2 mM EGTA and 2 mM MgSO_4,_ for MF20) and processed for whole-mount immunofluorescence staining as previously described (Ufartes et al., 2020). The *Xenopus* heart was visualized by staining against cardiac muscle troponin T (DSHB, CT3 antibody, dilution 1:30) or myosin heavy chain, sarcomere (MHC) (DSHB, MF20 antibody, dilution 1:100). Alexa Fluor 594 goat anti-mouse (Invitrogen, A-11005, dilution 1:400) was used as a secondary antibody.

### Whole-mount *in situ* hybridization

For the analysis of cardiac marker expression, embryos were injected with either Co MO or *fbrsl1* sp MO at the indicated concentrations, together with *lacZ* RNA as a lineage tracer in one dorsal blastomere at the four-cell stage. Embryos were fixed using MEMFA (3.7% formaldehyde, 0.1 M MOPS, 2 mM EGTA and 2 mM MgSO_4_), and β-galactosidase staining and *in situ* hybridization were performed according to standard protocols (Harland, 1991; Smith and Harland, 1991). The *Xenopus laevis* full-length *fbrsl1* (3609 bp) was amplified by PCR from *Xenopus laevis* cDNA using the primers 5′-ATGGATATTAAAACCAAACAACCAAGCAGG-3′ and 5′-TATCGTGCCTCCACTTCCTTAGGG-3′. The PCR product was cloned into the pCR-Blunt II-TOPO vector (Zero Blunt TOPO PCR cloning kit, Thermo Fisher Scientific), from which sense and antisense RNA probes were synthetized. The *fbrsl1* expression pattern, including sense controls at all documented stages, was analyzed using wild-type embryos. Histological sections of stained embryos were prepared as previously described (Breuer et al., 2020). The following cardiac marker probes were used for functional analysis: *nkx2.5* (Tonissen et al., 1994), *mhcα* (Gessert et al., 2008) and *isl1* (Kelly and Melton, 2000).

### Live-imaging of beating hearts

For phenotypic analysis, *Xenopus laevis* embryos were injected into one dorsal blastomere at the four-cell stage with 7.5 ng of the respective morpholino oligonucleotides in combination with 50 pg *mGFP*. For live imaging of the beating hearts, embryos at stage 44 were anaesthetized in 0.1×MBS (modified Barth's saline) containing 0.01% benzocaine and movies of the beating hearts were recorded. The heart size, the length and width of the OFT and the ventricular area at the time point of contraction were measured using ImageJ.

### Imaging and statistical analysis

Phenotypical documentation was performed using a Nikon stereo microscope (SMZ18) with a DS-Fi3 Nikon camera and NIS-Elements imaging software. For immunofluorescence imaging, Stellaris 8 Falcon (Leica Microsystems) with LAS X software was used. The investigators were unaware of the group allocation when they assessed the experimental outcome. Statistical analysis was conducted using one-way ANOVA followed by Tukey's post hoc test with GraphPad PRISM Software (*P≤0.05, **P≤0.01, ***P≤0.001, ****P≤0.0001).

### Western blotting

One-cell stage embryos were injected with 10 ng of each morpholino and 100 pg *fbrsl1_A-eGFP* RNA. Ten embryos (stage 20) per condition were lysed in NP-40 lysis buffer [50 mM Tris (pH 7.5), 150 mM NaCl and 0.5% (v/v) NP-40 containing 1× Complete Protease Inhibitor (Roche)]. The protein extracts were separated by 12% SDS-PAGE, transferred to a nitrocellulose membrane (AmershamProtran) by electroblotting and blocked in Intercept (TBS) Blocking Buffer (LI-COR) or TBS buffer [50 mM Tris-HCl (pH 7.5) and 150 mM NaCl] containing 5% nonfat dried milk. The following antibodies were used for detection of proteins: anti-GFP (Abcam, ab290, 1:1000) and anti-actin (Merck Millipore, MAB1501, 1:2000) antibodies. Tween-20 was added to the antibody solution at a final concentration of 0.2%. IRDye-conjugated secondary antibodies (LI-COR) were used: IRDye 800CW donkey anti-rabbit IgG secondary antibody (LI-COR, 926-32213, 1:7500) and IRDye 680RD donkey anti-mouse IgG secondary antibody (LI-COR, 926-68072, 1:7500). Proteins were detected using the Odyssey Fc Imaging System (LI-COR Bioscience).

## Supplementary Material

10.1242/dmm.050507_sup1 Supplementary information
