# Supplementary material for: Fbrsl1 is required for heart development in Xenopus laevis and de novo variants in FBRSL1 can cause human heart defects
Source: Dis Model Mech. 2024 May 14;17(6):dmm050507. doi: 10.1242/dmm.050507 (PMC11128277; doi:10.1242/dmm.050507)
Supplement: Supplementary information [file dmm-17-050507-s1.pdf]

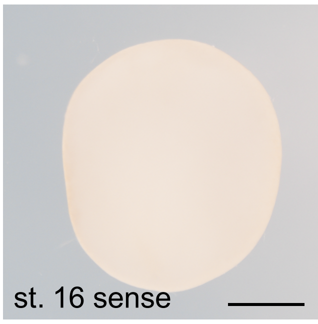

Fig. S1. A stage 16 embryo analyzed using a sense *fbrsl1 in situ* hybridization probe.

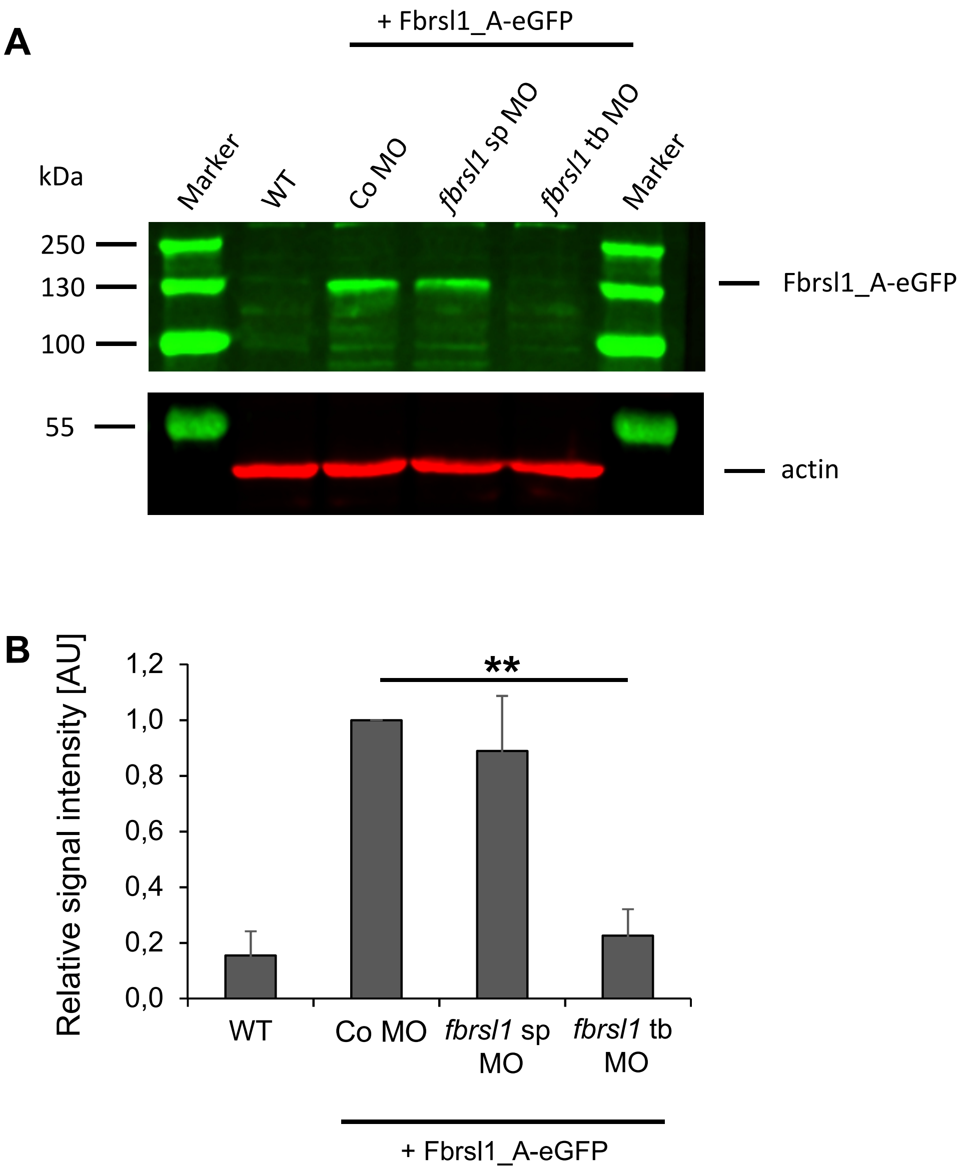

Fig. S2. *fbrsl1* tb MO efficiently inhibits the translation of a *Xenopus* GFP-tagged Fbrsl1 isoform (Fbrsl1_A-eGFP). A One-cell stage embryos were injected with 10 ng Co MO, *fbrsl1* sp MO or *fbrsl1* tb MO together with 100 pg *fbrsl1_A-eGFP* RNA, a shorter Fbrsl1 transcript, isolated from wild-type *Xenopus* embryos. Protein expression was analyzed by western blotting using an α-GFP antibody. The Fbrsl1_A-eGFP protein is detected in embryos injected with the *fbrsl1_A-eGFP* in combination with Co MO or *fbrsl1* sp MO. However, Fbrsl1_A-eGFP expression is strongly inhibited in embryos co-injected with the *fbrsl1* tb MO. **B** The ratio of signal intensity levels of Fbrsl1_A-eGFP to actin were normalized to the Co MO + Fbrsl1_A-eGFP levels and plotted. The graph shows the mean relative signal intensity (AU, arbitrary units) of Fbrsl1_A-eGFP expression of four independent experiments, s.e.m. are shown, $^{**}P < 0.01$ (one-way ANOVA).

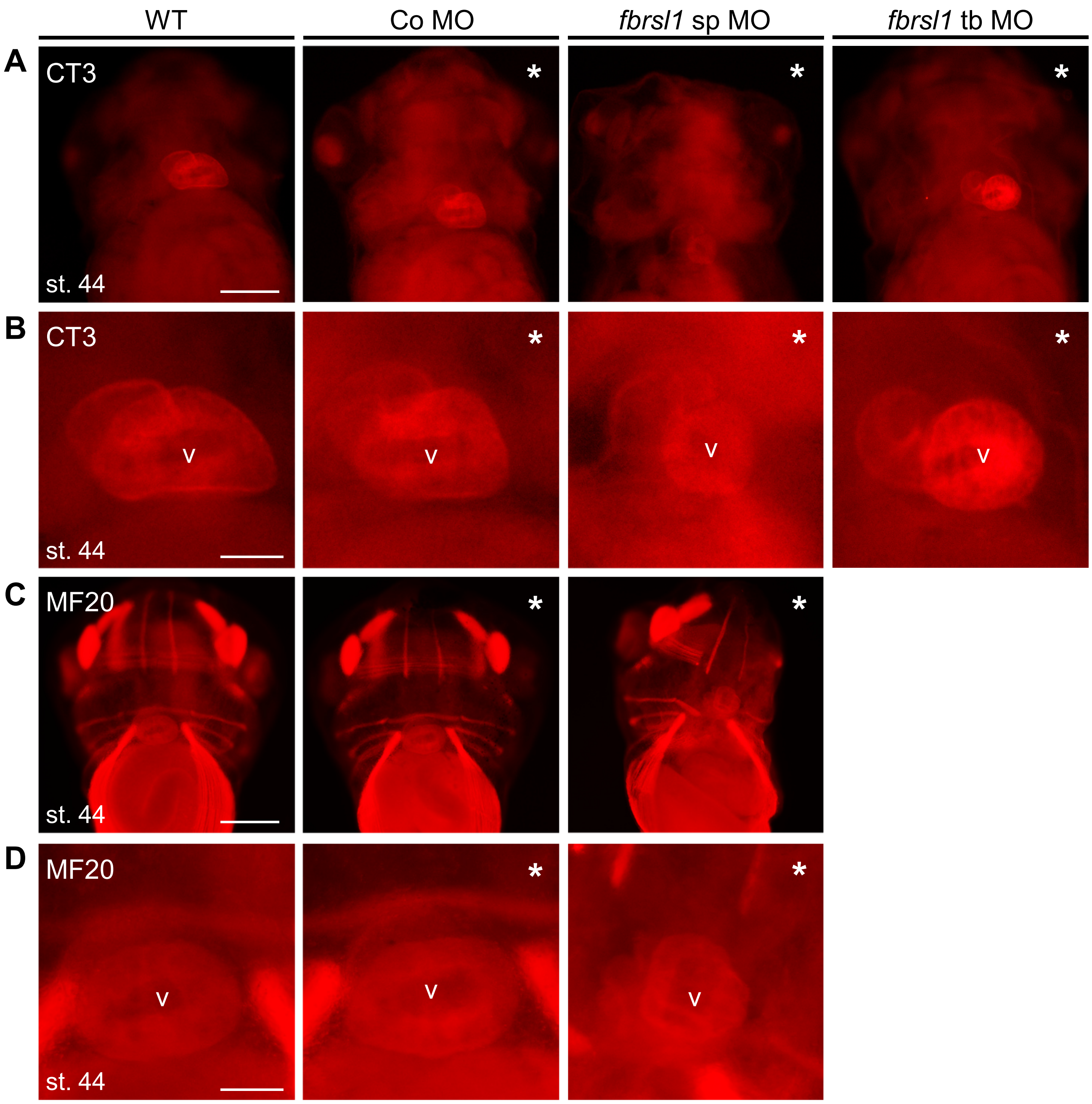

Fig. S3. Heart morphology analyzed by antibody staining. Embryos were injected in one dorsal blastomere at the four-cell stage with 7.5 ng Co MO, *fbrsl1* sp MO or *fbrsl1* tb MO and 50 pg *mGFP* RNA (CT3) or 100 pg *lacZ* RNA (MF20) as lineage tracer. **A, B** CT3 antibody staining. **C, D** MF20 antibody staining. **B, D** Higher magnification of the heart region shown in the images in **A** and **C**, respectively. Asterixs marks the injected side. Scale bar in **A** and **C** 500 µm, in **B** and **D** 100 µm. v, ventricle.

Disease Models & Mechanisms • Supplementary information

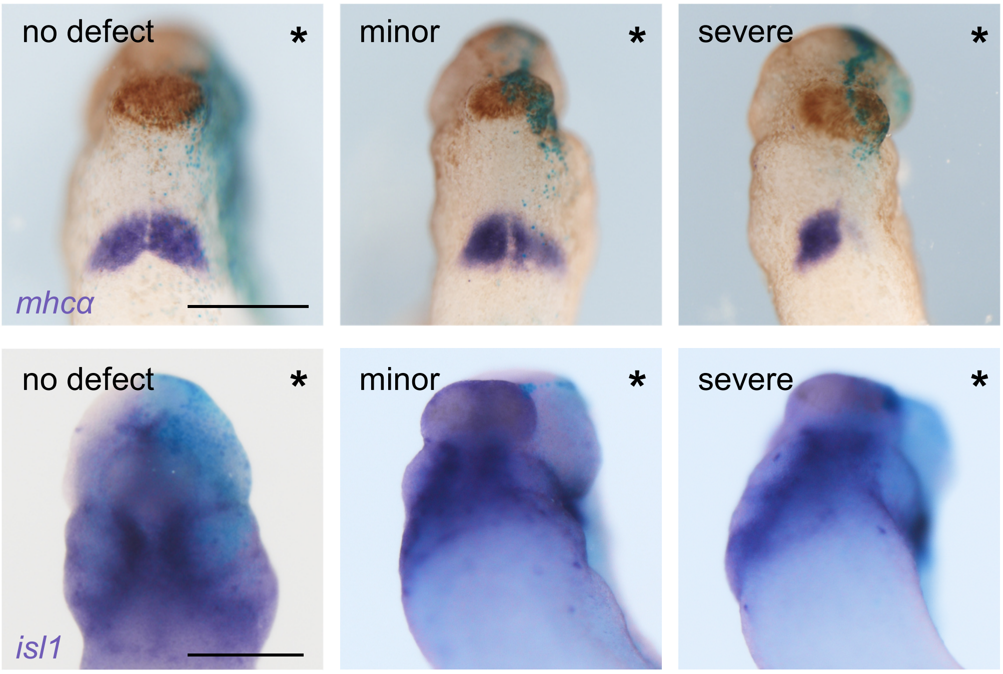

Fig. S4. Categorization of *mhcα* and *isl1* patterning defects. For statistical analyses of *mhcα* and *isl1* patterning defects, the embryos were categorized as having no, minor or severe defects. Representative embryos for each category are shown. Asteriks marks the injected side.

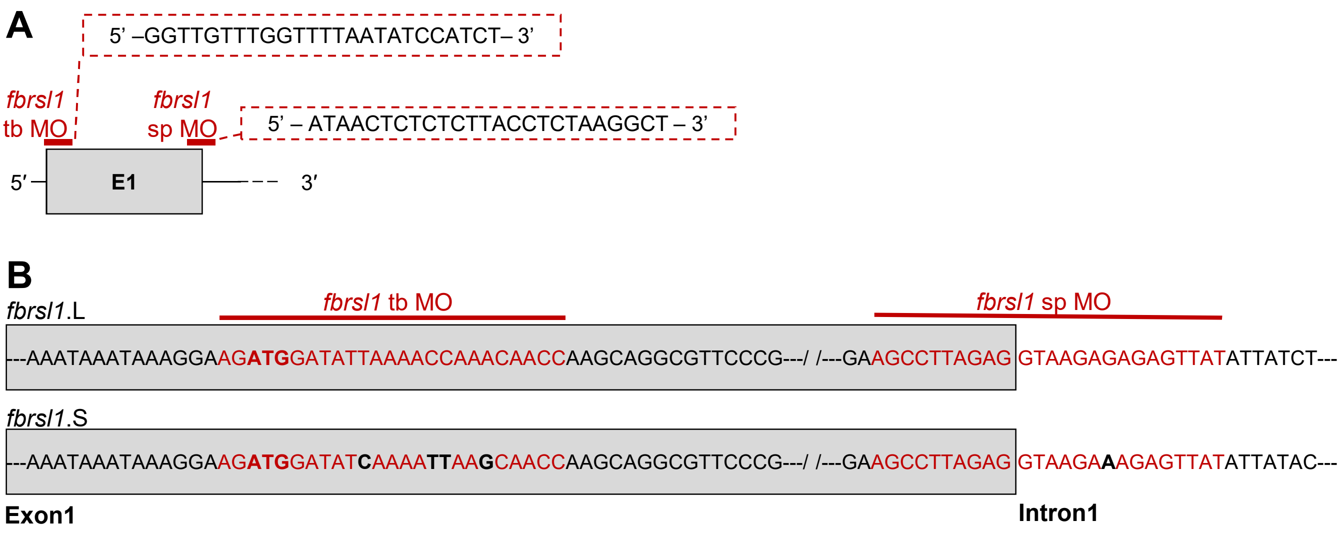

Fig. S5. *fbrsl1* sp MO and *fbrsl1* tb MO binding sites on *Xenopus laevis fbrsl1.L* and *fbrsl1.S*

A The schematic shows the *fbrsl1* tb MO and *fbrsl1* sp MO binding sites on exon 1 and the exon1/intron1 boundary. The corresponding morpholino sequences are indicated in the dashed square. **B** shows a section of the *fbrsl1.L* and the *fbrsl1.S* sequences with the corresponding morpholino binding sites marked. Both morpholino oligonucleotides were designed to target the *fbrsl1.L* form. The *fbrsl1* tb MO has 4 mismatches with *fbrsl1.S* and the *fbrsl1* sp MO has 1 mismatch. ATG triplet is shown in bold.

Disease Models & Mechanisms • Supplementary information

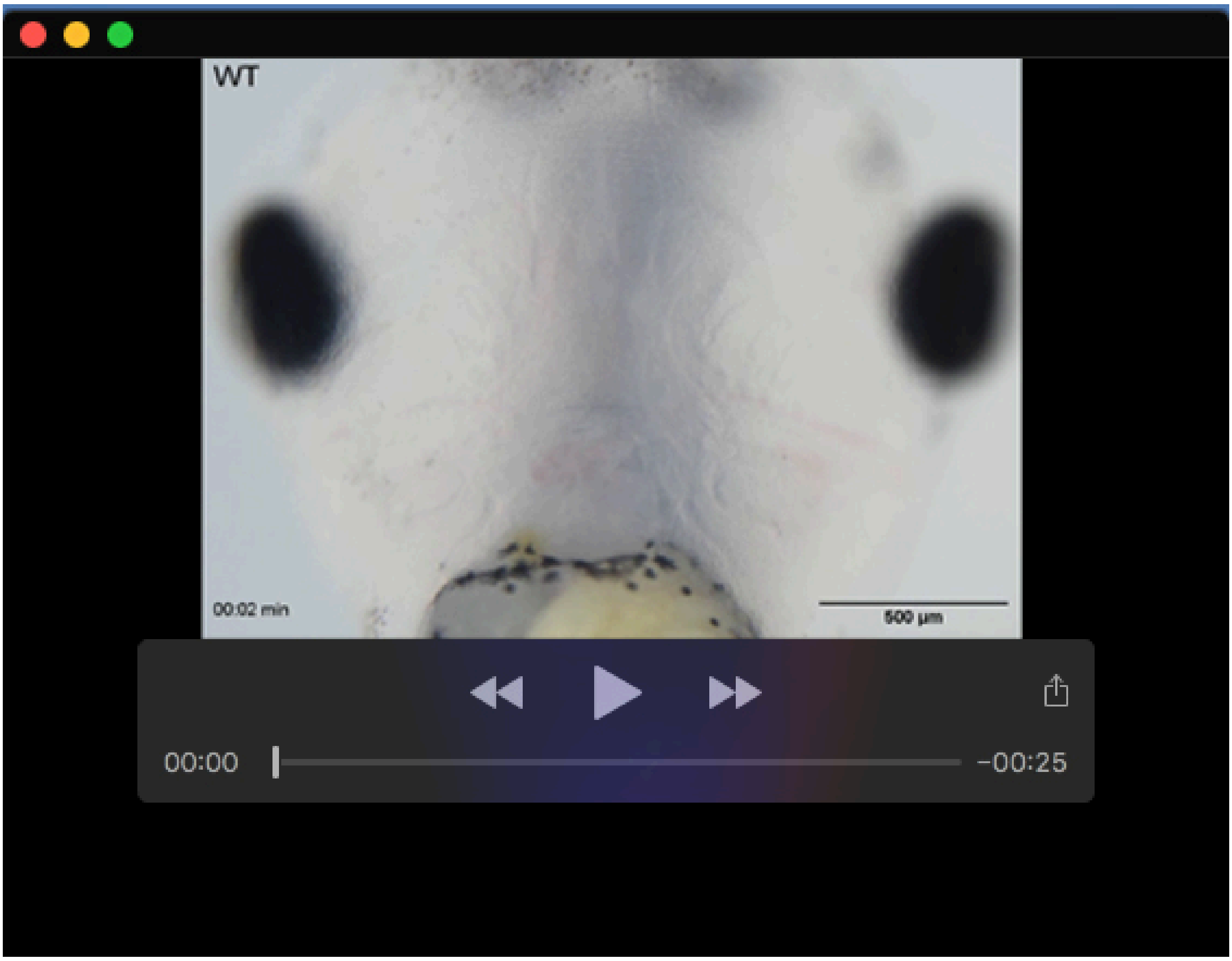

Movie 1. The beating heart of a wild-type *Xenopus laevis* embryo. Wild-type embryos show a dynamic beating of the heart and blood flow through the ventricle and outflow tract. A representative embryo is shown from the ventral side.

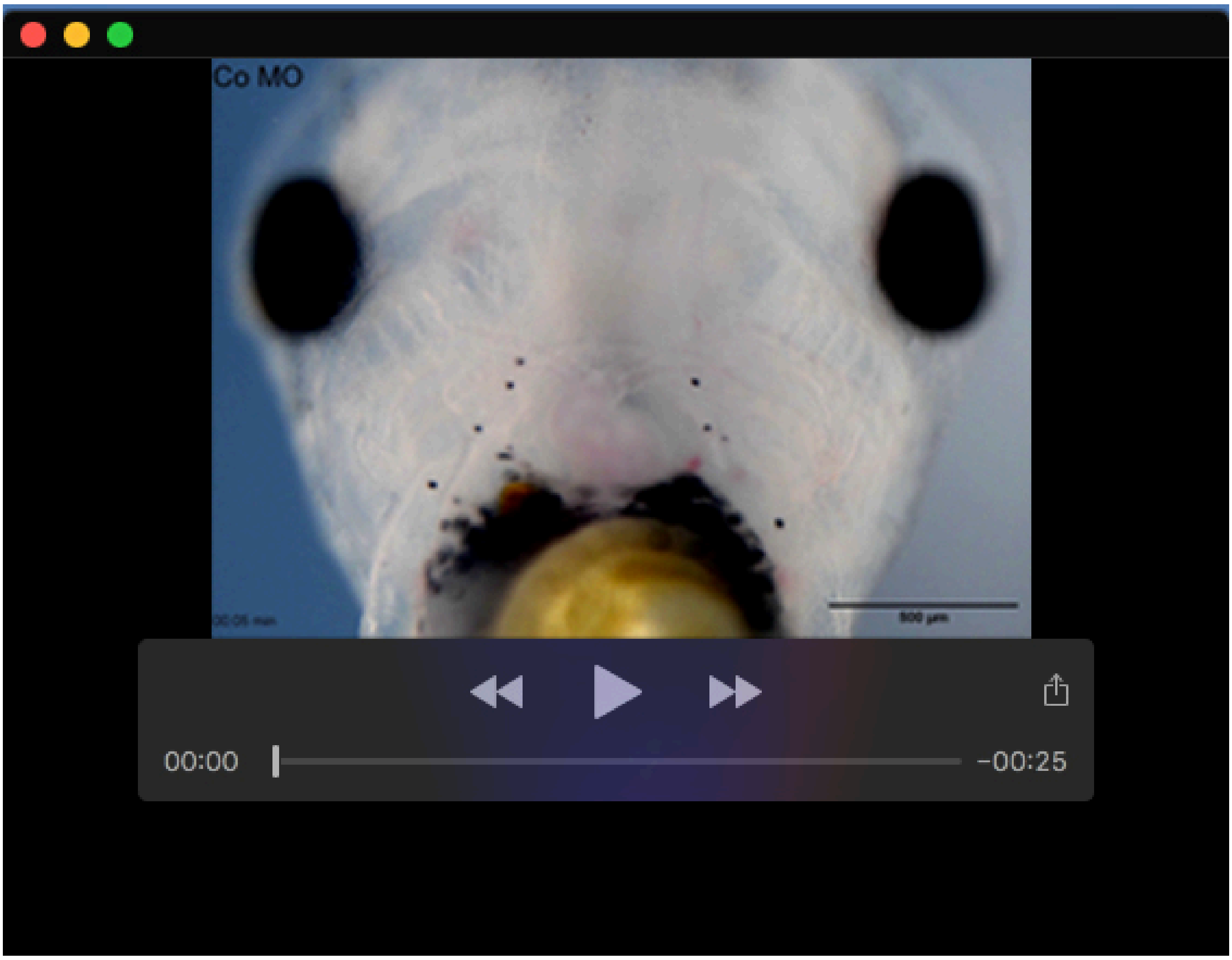

Movie 2. The beating heart of a *Xenopus* embryo injected with Co MO. Four-cell-stage embryos were injected in one dorsal blastomere with 7.5 ng Co MO in combination with 50 pg *mGFP* RNA as a lineage tracer. The embryos were anesthetized with 0.1x MBS (modified Barth's saline) containing 0.01% benzocaine. Beating hearts were recorded at stage 44. A representative embryo is shown from the ventral side. The embryo shows a dynamically beating heart; the blood flow through the ventricle and outflow tract is visible.

Disease Models & Mechanisms • Supplementary information

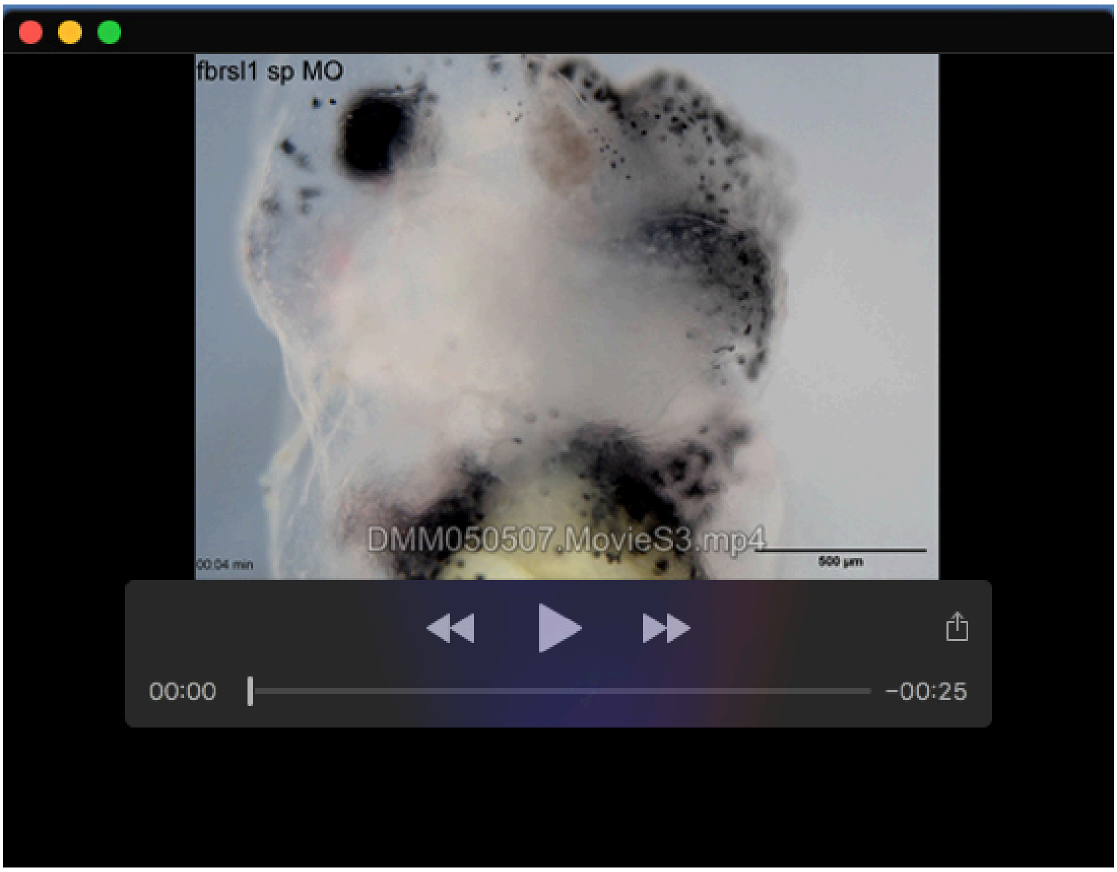

Movie 3. The beating heart of a *Xenopus* embryo injected with fbrsl1 sp MO. Four-cell-stage embryos were injected in one dorsal blastomere with 7.5 ng *fbrsl1* sp MO in combination with 50 pg *mGFP* RNA as a lineage tracer. The embryos were anesthetized with 0.1x MBS (modified Barth's saline) containing 0.01% benzocaine. Beating hearts were recorded at stage 44. A representative embryo is shown from the ventral side. The heart is malformed and has a smaller ventricle, but is able to beat. The blood flow is not visible.

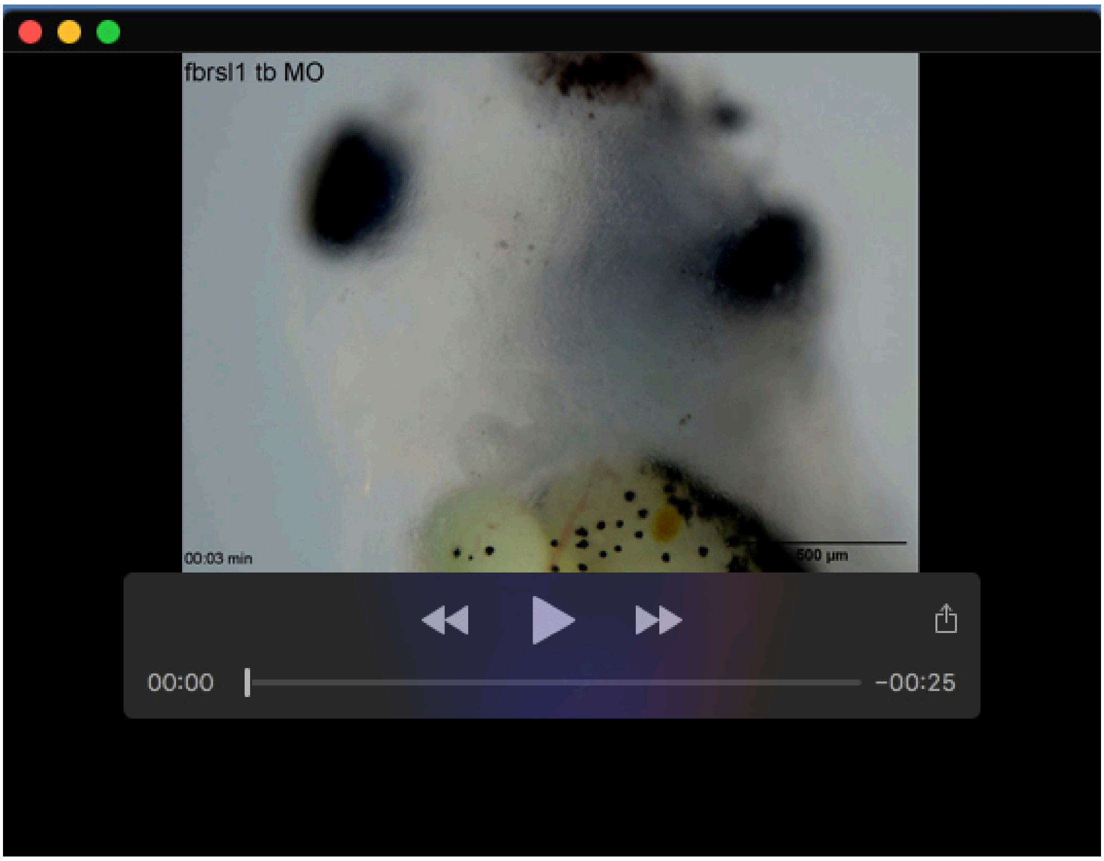

Movie 4. The beating heart of a *Xenopus* embryo injected with fbrsl1 tb MO. Four-cell-stage embryos were injected in one dorsal blastomere with 7.5 ng *fbrsl1* tb MO in combination with 50 pg *mGFP* RNA as a lineage tracer. The embryos were anesthetized in 0.1x MBS (modified Barth's saline) containing 0.01% benzocaine. Beating hearts were recorded at stage 44. A representative embryo is shown from the ventral side. The heart is severely malformed and the blood flow is not visible.

Disease Models & Mechanisms • Supplementary information
